# Use of a low-tech tool in the improvement of social interaction of patients with Rett Syndrome: an observational study

**DOI:** 10.3389/fpubh.2024.1353099

**Published:** 2024-04-04

**Authors:** Tindara Caprì, Lucia Dovigo, Martina Semino, Meir Lotan, Nasrin Mohammadhasani, Giuseppina Zamarra, Rosa Angela Fabio

**Affiliations:** ^1^Department of Life and Health Sciences, and Health Professions, Link Campus University, Rome, Italy; ^2^Airett Innovation and Research Center, Verona, Italy; ^3^Department of Physical Therapy, Ariel University, Ariel, Israel; ^4^Department of Educational Technology, Faculty of Educational Sciences and Psychology, Kharazmi University, Tehran, Iran; ^5^Fondazione Asphi, Bologna, Italy; ^6^Department of Economics, University of Messina, Messina, Italy

**Keywords:** Rett Syndrome, social interaction, low technology, rehabilitation, school setting

## Abstract

**Introduction:**

The main aim of the present study was to examine whether the use of a low-tech tool, called click4all, inserted into cognitive and motor training can increase social interaction of patients with Rett Syndrome (RTT) with classmates in a school setting.

**Methods:**

Twenty-seven participants with RTT were randomly assigned to two groups: the experimental group received treatment with click4all, and the control group received traditional treatment without click4all. Parameters were measured before treatment (T1), 6 months after treatment (T2), 6 months after the second treatment phase (T3) and at the end of the third treatment phase (T4).

**Results:**

The results demonstrated an increase in levels of social interaction among classmates and patients with RTT in the experimental group, over time, compared to the control group, 95% CI [5.20–15.30]. Classmates also showed a higher level of knowledge related to participants of the experimental group, and this increased over time, 95% CI [24.98–63.52]. The level of knowledge related to the control group was stable over time and lower than the experimental group.

**Discussion:**

This study demonstrated that the use of a low-tech tool can increase social interactions of patients with RTT in a school setting. This is important, as patients with RTT are often restricted in an isolation condition.

## 1 Introduction

Rett Syndrome (RTT) is a severe, neurodevelopmental disorder mainly caused by mutations in the MECP2 gene, affecting around 1 in 10,000 female births (1–3). Patients with RTT initially appear to follow a typical development path, but at about 18 months of age a subtle regression in developmental acquisitions begins, opening the path to the clinical stages (4, 5). Initially, there is an early onset deceleration stage, characterized by a psychomotor retardation of development with a deceleration in head growth and a noticeable reduction in the child’s play, communication, and social interaction. There is then a regression phase, rapid destructive stage, characterized by loss of previously acquired language skills and of purposeful hand use, increasing difficulties in motor abilities and intellectual disability, gross motor skills are also affect-ed with delayed walking and gait abnormalities (5). After the regression phase, in the third pseudostationary stage, the disorder is mainly stable, but seizures, ataxia, and scoliosis appear. The last phase, by about 10 years of age, is the final late motor deterioration stage, scoliosis worsens, and mobility is often so severely limited that most patients will require the use of a wheelchair (6–9).

The impaired clinical picture typical of RTT makes it difficult to identify successful rehabilitation strategies. Literature shows that high frequency and low intensity rehabilitation leads to an improvement and increase in performance in all areas, from motor to cognitive aspects (10–18). It has also demonstrated that the involvement and collaboration of families and caregivers is an important factor for the success of treatment programs (19).

However, no study has investigated the involvement of other significant people, such as classmates of patients with RTT, in treatment programs. It is essential to re-member that patients with RTT are often confined to a passive and isolated condition since they have difficulty interacting with objects, engaging in recognized forms of communication, or controlling stimulation (20). For this reason, the involvement of classmates of patients with RTT in social treatments could be an option available to clinicians and parents in providing positive environmental stimulation (21).

With reference to treatments for RTT, it has been demonstrated that patients with Neurodevelopmental Disorders (NDDs), including RTT, can benefit from the use of technology-aided programs, virtual reality-based rehabilitation, eye gaze digital games, and technology-based therapy; these technologies can help improve cognitive, motor, and behavioral skills. Moreover, it was found that the use of these technologies or low-tech tools can stimulate social interaction between patients with NDDs and their classmates, increase learners’ motivation and social passages of their classmates (22, 23).

With reference to the use of low-tech tools in RTT, literature is still limited (24), some studies have used software for Augmentative and Alternative Communication aimed to improve communication abilities of patients with RTT and to make requests and/or initiate social-communication interactions. Other studies have used micro-switches devices linked to computer systems that can be activated with small responses (small head and hand movements) by patients. Overall, these studies have provided positive and significant results, but no study involved classmates of patients with RTT in the therapeutic interventions.

Given the potential benefits of using technology in social and school settings, it is becoming essential to understand how technology can offer better opportunities for treatment of cognitive and motor deficits in RTT and, consequently, provide the best approach toward the goal that is set. To explore the above-mentioned issue and to also investigate the role of the involvement of other significant people in treatment programs for RTT, the main aim of the present study was to examine whether the use of a low-tech tool, called click4all, inserted in a cognitive and motor training program, can increase social interaction of patients with RTT with classmates in a school setting.

Overall, click4all is a self-build kit and is configured as an interface for sensors. It is used to allow people with disabilities access PC, smartphone, and tablet technology, through interfaces built and customized to their cognitive, motor and sensory skills and abilities. Hence, the due to ease use, the possibility of customizing and the theoretical background of the click4all, we believe that this low-tech tool can play role as a facilitator for social interactions of patients with RTT.

In this study, social interaction was examined in two conditions (training program with and without click4all) over a 2-year period and considering both objective and subjective assessments. More in depth, to fully understand social interactions, recent studies suggested (25) the importance of differentiating between objective and subjective aspects of social interactions. Objective aspects are quantifiable but that take away individual perspective, for example number of eye contact, percentage of time spent alone. Subjective aspects are more experiential and customized, for example how individuals subjectively experience their interactions with others. In the present study, we have considered both aspects of social interactions, in terms of objective aspects we have examined the number of social episodes among classmates and patients with RTT; in terms of subjective aspects, we have examined classmates’ social ideas and knowledge about their classmate with RTT.

Given that the use of technology encourages social interaction and stimulates social skills (26, 27), we expected an increase in the level of social interaction in the group with click4all compared to the group without click4all. In both groups the interaction was initiated by teacher, who carried out the training sessions; the classmates and patients with RTT, in random turn, performed tasks requested in both training. Given that click4all aimed to stimulate social interaction, with no clinical therapeutic purposes, we expected similar improvements in cognitive and motor abilities in both groups.

## 2 Materials and methods

### 2.1 Participants

Twenty-seven patients with a diagnosis of RTT, ranging from age 5 to 38 years old (mean age 15.86 ± 11.27 years) were recruited from within the Italian Rett Association (AIRETT). Patients with RTT were classified as clinical stage III (characterized by prominent hand apraxia/dyspraxia, preserved ambulation ability, and some communicative ability, mainly eye contact) or stage IV (late motor deterioration, with progressive loss of ambulation ability), according to the criteria for classic RTT by Hagberg (28). Their grade of schooling was Italian kindergartens and higher levels of schools. A general assessment was conducted by a psychologist before starting the experimental sessions, using Downs’ scale (29) for evaluating the level of purposeful hand function and the Rett Assessment Rating Scales (RARS) (30) to evaluate the severity of the disease in patients with RTT. The Mecp2 mutation was seen in 100% of the sample; patients with FOXG1 and CDKL5 were excluded from the sample.

Inclusion criteria were female patients with genetically confirmed RTT who were between 5 and 38 years old and at III or IV clinical stage (without assistance at the time of their enrolment). Exclusion criteria were FOXG1 and CDKL5 mutation and presence of comorbid non-Rett–related disease.

Participants were matched for age, severity level of the disease, and functional ability level and randomly assigned to two groups: the experimental group received the treatment program with click4all, and the control group received traditional treatment without the use of click4all. Table 1 shows the characteristics of the groups.

**Table 1:** Characteristic of participants with RTT.

Groups	Participants	Clinical stage	Age	RARS total score^1^	Down’s Scale total score^2^
Group with click4all	1	IV	31	71	2
	2	III	18	66	3
	3	III	5	86	1
	4	III	6	71	1
	5	III	7	64	5
	6	III	6	66.5	2
	7	IV	38	86	2
	8	III	5	64.5	1
	9	III	12	63	1
	10	III	8	70	1
	11	III	5	56	3
	12	III	12	71.5	1
	13	III	5	65.5	4
	14	III	10	53.5	3
	15	III	6	86	1
Group without click4all	1	IV	31	58	2
	2	III	18	65	1
	3	III	5	86	1
	4	III	6	57	3
	5	III	7	63	3
	6	III	6	64	3
	7	IV	38	65.5	2
	8	III	5	70	5
	9	III	12	61	2
	10	III	8	59	1
	11	III	10	75.5	2
	12	III	12	75.5	1
				

^1^Rett Assessment Rating Scales (RARS). ^2^Down’s Scale for the level of purposeful hand functioning.

Fifty-four typically developing subjects (15 men and 35 women), between 6 and 25 years (M = 14.93, SD = 2.53), were randomly recruited from classmates of each patient with RTT. They were randomly involved in the training sessions. These participants were Italian and have no psychological or neurological disease. Their grade of schooling was Italian kindergartens and higher levels of schools. Inclusion criteria were male and female with typical development who were between 6 and 30 years old. Exclusion criteria were presence of neurological, motor and psychiatric disease.

As the study was conducted for the period of 2 years, we specify that some participants missed some sections due to family and/or personal reasons. However, the number of missing sections was very small over 2 years (from 1 to 3).

### 2.2 Study design

**Figure 1:**
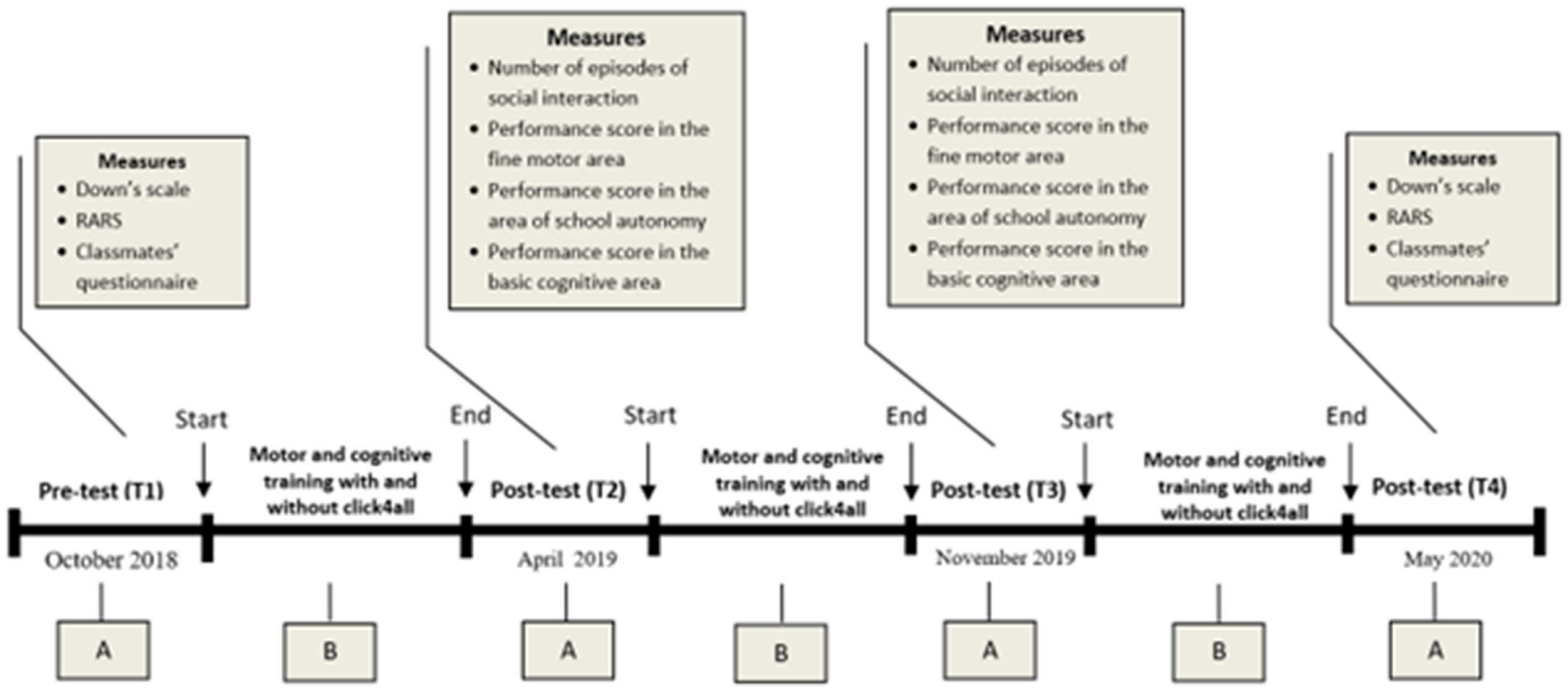
Experimental design. This study lasted two years (from October 2018 to end of May 2020). It started with the baseline assessment of participants with RTT (T1, First A). From November 2018 to March 2019, motor and cognitive training with and without click4all were carried out (First B). In April 2019, the post-test assessment of participants was carried out to test change after treatment (T2, second A). From May 2019 to October 2019, the training was again applied (Second B). In November 2019, the post-test assessment of participants was again carried out (T3, third A). From December 2019 to April 2020, the training was again applied (Third B); In May 2020, the post-test assessment of participants was carried out (T4, fourth A).

This study employed an experimental design ABABABA. It represented an attempt to measure baseline in pre-test phase (the first A phase), treatment measurement (the first B phase), withdrawal of treatment and measuring the change (the second A phase), re-introduction of treatment (the second B phase), again measuring the change (the third A phase) and then again applying the treatment and measuring the change with the post-test phase (the third B phase and the fourth A phase; see Figure 1).

In the pre-test phase and the last post-test phase, all participants underwent a global evaluation through use of the RARS and Downs’ scales. Moreover, in all post-test phases and in the pre-test phase, the number of episodes of social interaction among participants with RTT and their classmates was measured. In these phases, the therapist also evaluated: the level of social knowledge of classmates, the level of performance of hand motor skills, and the level of cognitive performance in the recognition of basic cognitive concepts of all participants with RTT.

In the three B phases, all participants received 30 min of motor and cognitive rehabilitation for 3 days a week over a 4-month period at school in a laboratory setting in a small group with classmates. The experimental group received the treatment using the click4all tool, whereas the control group received traditional cognitive and motor treatment.

All the assessment phases were video recorded. Two independent blind observers, blinded to the study aims, design, hypotheses, analyses, and outcomes, coded each video to evaluate the parameters. They were psychologists and therapists with extensive experience in behavioral coding of patients with RTT. Inter-rater reliability was calculated using Cohen’s Kappa which showed excellent overall agreement among raters (k = 0.80, *p* < 0.001).

### 2.3 Instruments for global evaluation

As mentioned above, at the beginning and end of the study, global assessments were carried out to identify the profile of patients with RTT through the following scales and questionnaires. RARS (30) is a standardized scale used to evaluate patients with RTT. It is organized into seven domains: cognitive, sensorial, motor, emotional, autonomy, typical characteristics of the disease and of behavior. Typical characteristics of the disease and behavior domains measure the following characteristics: mood swings, convulsions, dyspnoea, hyperactivity, anxiety, aggressivity, bruxism, oculogyric crises, epilepsy, aerophagia, muscle tension and food preferences. A total of 31 items was generated as representative of the profile of RTT. Each item is provided with a brief glossary explaining the meaning in a few words. Each item is rated on a 4-point scale, where 1 = within normal limits, 2 = infrequent or low abnormality, 3 = frequent or medium-high abnormality, and 4 = strong abnormality. Intermediate ratings are possible; for example, an answer between 2 and 3 points is rated as 2.5. For each item, the evaluator circles the number corresponding to the best description of the patient. After a patient has been rated on all 31 items, a total score is computed by summing the individual ratings. This total score allows the evaluator to identify the level of severity of RTT, conceptualized as a continuum ranging from mild symptoms to severe deficits (Mild = 0–55; Moderate = 56–81; Severe ≥ 81). RARS was established by a standardization procedure, involving a sample of 220 patients with RTT, proving that the instrument is statistically valid and reliable. More precisely, normal distribution analyses of the scores were computed, and the mean scores of the scale were similar to the median and the mode. Skewness and kurtosis values, calculated for the distribution of the total score were 0.110 and 0.352, respectively. Distribution was found to be normal. Cronbach’s alpha was used to determine the internal consistency for the whole scale and sub-scales. Total alpha is 0.912, and internal consistency of the sub-scales was high (from 0.811 to 0.934).

Downs’ scale for level of purposeful hand function (29) is a scale that defines the level of motility of the hands of patients with RTT by assigning a score from 1, the minimum of manual functionality, to 8, the maximum of manual functionality; in particular the score is given: (1) No observed hand function; (2) Able to hold at least one large object (cup, spoon, small ball or toy) > 2 s; (3) Assistance to grasp but able to pick up and hold at least one large object > 2 s; (4) Able to grasp., pick up, and hold at least one large object > 2 s; (5) Able to grasp., pick up, and hold at least one large object > 2 s and use a raking grasp to grasp., pick up and hold a small object (e.g., sultana, sweet, or small piece of sandwich) > 2 s; (6) Able to grasp., pick up, and hold at least one large object > 2 s and use the radial side of the hand to grasp., pick up, and hold a small object > 2 s (can be a scissors, inferior pincer, or superior pincer grasp); (7) Skills for level 6 and able to transfer an object from one hand to the other (accurate preshaping of the hand is not seen); (8) Skills for level 7 and when hand is approaching an object, hand orientation and size recognition closely approximates the position and size of the object.

### 2.4 Measures

The following parameters were evaluated every 6 months:

1. Number of episodes of social interaction. To measure the social impact of the study within the school, the average number of social episodes among classmates and patients with RTT was calculated. To define social episodes, we observed the following behaviors: eye contact, smile and physical contact. The observation was carried out during the first 10 min of free time in the school.
2. Classmates’ social ideas and knowledge. A questionnaire was structured to investigate the level of knowledge of classmates about the preference of participants with RTT. This questionnaire consisted of 5 questions with closed responses (yes or no). Three questions investigated favorite cartoon, color, and song and two questions investigated the idea of classmates about the potential learning of participants with RTT. For example, question 1 was aimed to assess the favorite cartoon, as follows: “Do you know what her favorite cartoon is? If it is yes, define it.” Question 4 was aimed to assess the idea of potential learning, as follows: “Do you understand if something she likes or not? If it is yes, define it” (see Supplementary material). The questionnaire was administered by the teacher to each pupil. Each correct response was scored with 2 points. Correct response means that the pupil’s answer was exactly the favorite cartoon, song, color, way of expressing preferences of participants with RTT.
3. Performance score in the fine motor area. The participants were required to carry out four basic motor exercises, as follows: (1) reach for a tablet, (2) touch a tablet screen, (3) grasp a favorite game, and (4) bimanual coordination to hold a puppet with both hands. The participant sat at a table. The object to be reached was placed on the table, on the midline, and at a distance corresponding to half the length of the participant’s arm. The target size was equal to the palm size of the patient’s hand for mono hand skills and 10 cm for bimanual skills. The dominant hand was held on the table at the bottom edge, the non-dominant hand was kept gently blocked under the table (except in the bimanual coordination exercise, where both arms were on the table). After the object was shown enthusiastically to the participant, the examiner explained and showed the requested action. This procedure was carried out 5 times per exercise; the time allowed for the participant to activate her arm was 10 s, after which the request was interrupted. A score was given in relation to the autonomous performance of the activity: (1) if the patient never performed the exercise independently; (2) if the patient performed the exercise autonomously at most 2 times; (3) if the patient performed the exercise autonomously 3 times; (4) if the patient performed the exercise autonomously 4 times; (5) if the patient performed all 5 tests correctly and autonomously. A score of 0.5 was added if the patient showed constant eye coordination during the exercise.
4. Fine motor skills in school achievement. The participants were required to use a pen in four different tasks, as follows: (1) reaching for the instrument, (2) grasping the instrument placed on the bench, (3) maintaining a grasp of the instrument and (4) releasing the instrument on the bench in a controlled manner. The pen was placed on the table, on the midline. The dominant hand was gently held on the table at the bottom edge, the non-dominant hand gently kept blocked under the table. Then, the pen was shown enthusiastically to the participant, the examiner explained and showed the requested action. This procedure was carried out 5 times per exercise. The time allowed for the participant to activate her arms was 10 s, after which the request was interrupted. The following score was awarded: (1) if the patient never performed the exercise autonomously; (2) if the patient performed the exercise autonomously at most 2 times; (3) if the patient performed the exercise autonomously 3 times; (4) if the patient performed the exercise autonomously 4 times; (5) if the patient performed all 5 tests correctly and autonomously. A score of 0.5 was added if the patient showed constant eye coordination during the exercise.
5. Basic cognitive area. To measure basic cognitive skills, discrimination tasks were used, as follows: the participant had to choose the target stimulus between two different stimuli of the same semantic category. The participant sat at a table and had two stimuli of the same category positioned on the table. She was asked to look at them and to choose the target by looking at it and touching it (eye-hand coordination). If the participant did not have the motor ability to touch, only the eye response was considered. Then the position of the stimuli was changed, and the entire procedure repeated three times with the stimuli in random order (right, left and vice-versa). This procedure was repeated for each target of the category. The therapist presented all the target stimuli (5) and registered all the responses of the participants: each target was requested 3 consecutive times and, only if the participant performed all 3 correct answers was the target noted as already acquired. For each category, a score was given in relation to the correct and autonomous execution of the activity: (1) if the patient did not recognize any target, (2) if the patient recognized at most 2; (3) if the patient recognized 3; (4) if the patient recognized 4 concepts; (5) if the patient recognized more than 5 concepts. A score of 0.5 was added if the patient showed constant eye coordination during the exercise.

### 2.5 Click4all

Click4all was founded in 2013 based on experiences gained by ASPHI (Association for the development of digital projects for the disabled) in the field of digital accessibility for children, adolescents and adults with complex motor and cognitive disabilities. Click4all is a self-build kit and is configured as an interface for sensors, classified in the SIVA Portal of Aids (Accessories to Input Systems—ISO 22.36.15: Sensor Interfaces—SIVA 22.36.15.S02). It is used to allow people with disabilities access PC, smartphone, and tablet technology, through interfaces built and customized to their cognitive, motor and sensory skills and abilities. The click4all is extremely handle, light and resistant, therefore it is easily usable in school contexts.

**Figure 2:**
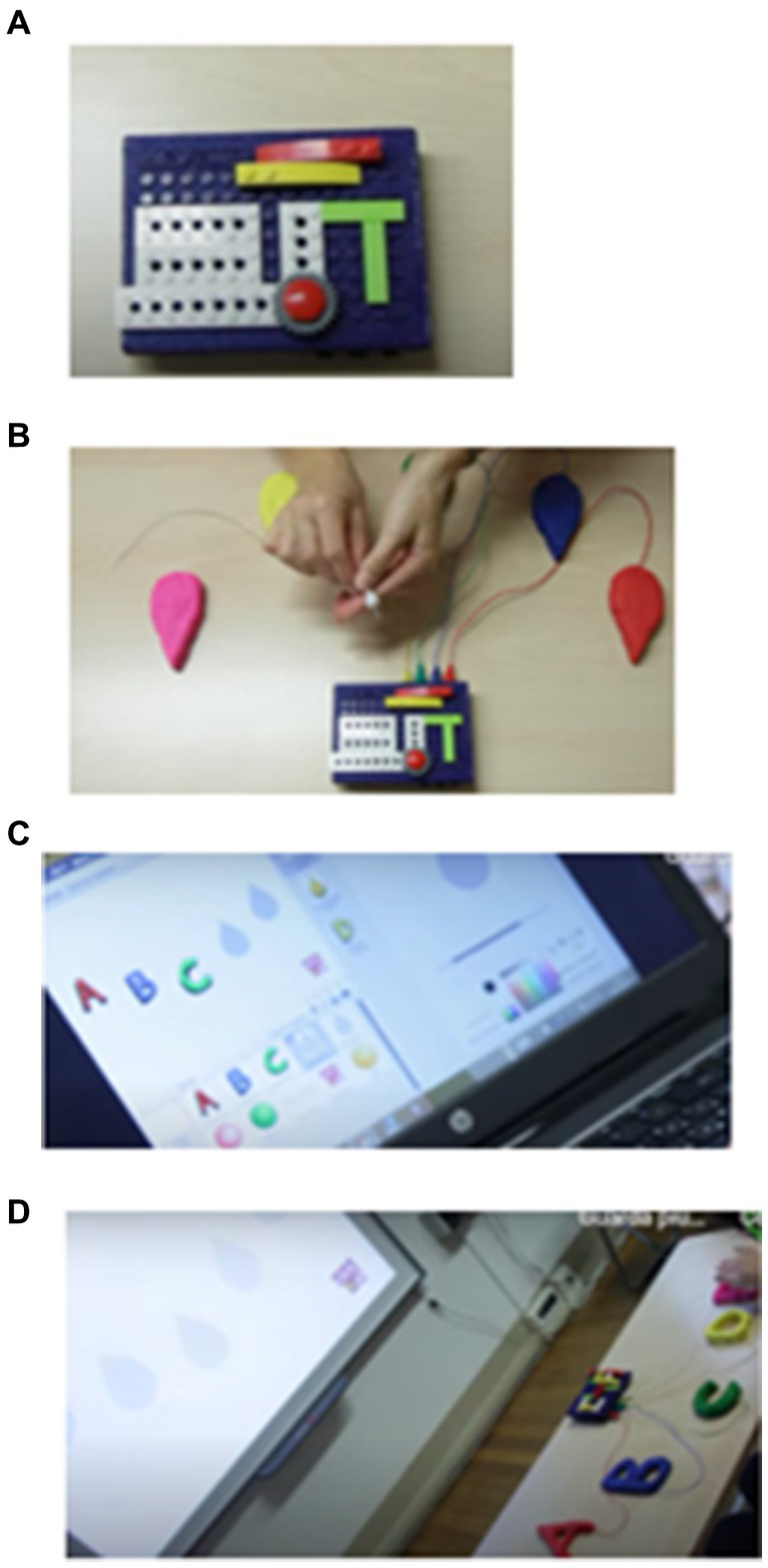
Click4all. Sequence of images depicting the operation of CLICK4ALL: panel **(A)** represents CLICK4ALL, panel **(B)** represents the conductive cables connecting CLICK4ALL to the material, panel **(C)** represents the preparation of the activity on the computer using Scratch, and panel **(D)** represents the connection between the various components and the initiation of the activity.

The click4all has a rectangular structure of 15 × 10 cm with a thickness of 3 cm; its surface is covered in Lego bricks so that the subjects can personalize it moving the bricks of click4all or using own Lego bricks (Figure 2A). In a side of this tool, there are some connect ports in which you can insert the sensors (Figure 2B). It is possible to create up to 18 custom buttons; in this study, 6 click sensors can be activated by pressing and 12 touch sensors are activated with a simple touch. Pressure can be adjusted according to the patient’s potential. Touch sensors can be created using conductive objects and materials (e.g., fruit, foil, metals, fabric and conductive ink).

At programming level, the click4all is integrated with Scratch a “graphical and free programming environment, created by MIT Boston,” designed to allow coding activities starting from primary school (Figure 2C). It is a graphic programming language, inspired by the constructionist theory of learning and very suitable for pedagogical projects for the stimulation of digital creativity and the development of computational thinking.

Scratch is a simple, free programming environment that uses a block-based, graphic, visual programming language. It is not necessary to type any code, as in traditional programming, but simply drag blocks of pre-set code, divided by color, inside the coding area and join them together in a logical order. It also allows people who have never programmed to intuitively create stories, animations, and games. Scratch games and activities have been created for single patients, and with it you can program interactive stories, games, animations, and activities that can be remixed and shared with other registered members of the community. The use of Scratch as an alternative tool in the activities of play, rehabilitation, educational recovery, can have a significant value. As written above, the clicl4all can be connected to different technological devices, in this study, the click4all was connected to a computer via Bluetooth or via USB (Figure 2D).

Before the start of experiment, an AIRETT therapist introduced to teachers the scratch games and activities and explained them the use of clik4all. An example of motor activity is the “grasping the marker” task, the teacher used a sensor that detects the contact of the participant’s hand with the marker. Once the contact was detected, a video of the marker drawing and a cartoon song were played on the computer screen. This encouraged the participant to interact with the marker and maintain contact. An example of cognitive activity is the “colour recognition task,” the teacher activated two sensors each linked to a specific color, when the participant’s hand touched one of the sensors, the corresponding color appeared on the screen together with a song. This helped the participant associate the tactile sensors with visual colors and learn color recognition concepts in a fun and engaging environment. This combination of touch sensors, visual and musical stimuli created an interactive and technological environment that can be effective in stimulating the participant’s involvement and social interactions between the classmates and patients with RTT during motor and cognitive training with click4all.

**Figure 3:**
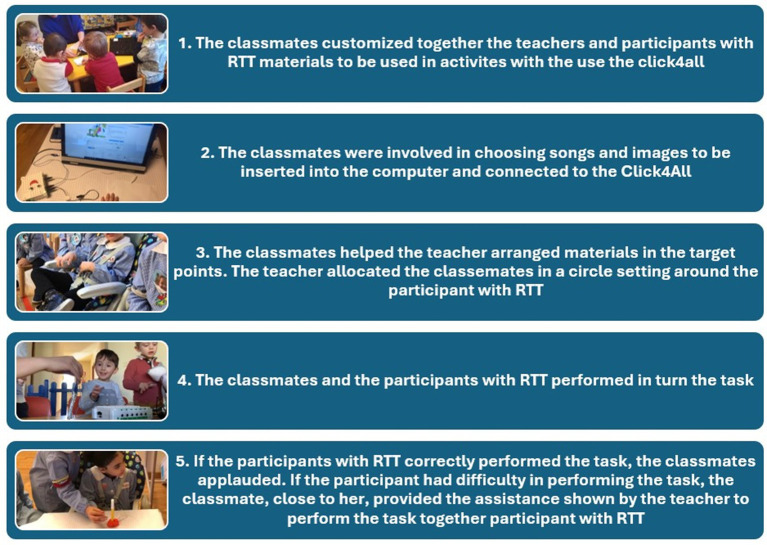
Sequence of involvement of classmates in the customizing of the device to work for tasks.

The classmates were involved in the creation of motivating and attractive targets to be used during the training as follows (Figure 3): together with the teacher, they chose the visual and auditory stimuli that should appear on the computer; for example, for the “grasping the marker” task, the classmates created a ball of Play-Doh in which the marker was inserted. Instead, for example, in the “recognising colours” task, the classmates colored two pictures of the participant’s favorite cartoon character they chose the pictures and songs of the character to be inserted into the computer.

### 2.6 Motor and cognitive and training with and without click4all

**Figure 4:**
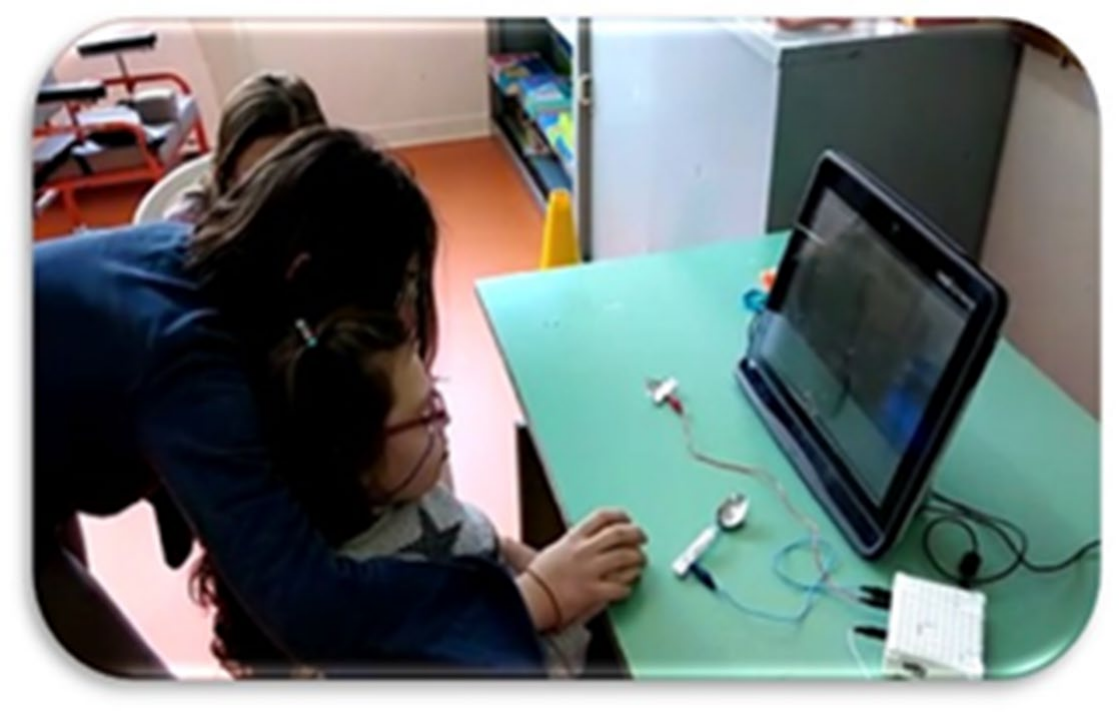
Training setting.

Both training sessions were carried out at school 3 times a week for 30-min over a 4-month period. All participants were tested in a quiet room of the school within school hours. This classroom seating arrangement consisted of a table and three chairs. The teacher was standing, and all participants (patients with RTT and two classmates) were sitting around a single large table (Figure 4). In both groups the interaction was initiated by teacher, who carried out the training sessions; the classmates and patients with RTT, in random turn, performed tasks requested in both training.

For the experimental group, each time a task was performed correctly the screen that was connected to click4all activated a visual and auditory animation customized for each activity for each participant. For the control group, when the task was correctly performed, verbal stimuli of teacher were presented (i.e., “Good job”).

In the training with click4all, the computer was positioned in front of participants. The dominant hand was held on the table at the bottom edge, the nondominant hand kept gently blocked under the table (except for the bimanual coordination exercise, where both hands were on the table). Then, the object was shown to participants on the screen of a computer or on the table, according to the type of training, and the teacher told the girls to perform an action. The teacher allowed 10 s to the participants with RTT to perform the action autonomously; after this time, the teacher gave the necessary help or proposed the request again. If the participant performed the task autonomously or with the indicated level of aid, the teacher gave her reinforcement, if the girl did not perform in a correct way, the teacher did not give any reinforcement and started a new session. The fixed criteria for each phase of the task were 3 correct consecutive answers for 3 consecutive sessions. Once the child learnt a movement, it was possible to improve the level of difficulty of skills. The girl’s performance was judged as follows: “+” = if the girl performed the task autonomously; “+−” = if the girl performed the task with aid; “−” = if the girl did not perform the task.

### 2.7 Motor training for the use of scholastic tool

The teacher prepared a pencil or a marker. The participant with RTT sat at the table. A pen was placed on the table, in a vertical position near the hand of the girl. The dominant hand was held on the table at the bottom edge, the non-dominant hand kept gently blocked under the table. The teacher explained and showed the request action. The teacher allowed 10 s to the participant to perform the movement autonomously, after this time the teacher gave the necessary help or proposed the request again. If the participant performed the task autonomously or with the indicated level of aid, the teacher gave her reinforcement, if the participant did not perform in a correct way, the teacher did not give any reinforcement and started a new session. The fixed criteria for each phase of the task were 3 correct consecutive answers for 3 consecutive sessions. Once the participant learnt to take it, it was necessary to increase the grip time; once this ability was acquired, the marker was placed horizontally on the table. In order to facilitate gripping, it may be useful to use the gripping supports which facilitate this movement. The participant’s performance was judged as follows: “+” = if the participant performed the task autonomously; “+−” = if the participant performed the task with aid; “−” = if the participant did not perform the task.

The aid in motor exercises is a gradual physical aid that goes from most to least, or from a more intrusive physical help to a less intrusive one. The first teaching step is to request approach to the object with total help from the hand, the second step with help from the wrist, the third step from the elbow and the fourth from the shoulder and finally completely fade the help. The transition from one level of aid to another occurs when the participant performs 3 correct consecutive answers for 3 consecutive sessions with that level of aid.

### 2.8 Cognitive training for basic concepts

Cognitive training was based on three cognitive-behavioral strategies: imitation procedures, prompting, generalization. The goal of this training was to develop basic cognitive skills, such as discrimination and recognition of common objects (food, toys and familiar objects). The stimuli consisted of 40 colored pictures of common objects with a dimension of 10 × 15 cm. The setting was individual. Patients with RTT sat at a table and had the target stimulus on their right and a distractor on the left. They were asked to look at them both and to choose the target by looking at it or touching it (if movement skills were good). The target stimulus was presented five consecutive times, the position of the stimuli was changed in a random order (right–left and vice versa). When the patient recognized the correct picture for three consecutive times, according to scientific consensus (12, 15) the object was recognized and learned. Thus, another target stimulus was presented with the same procedure.

### 2.9 Statistical analysis

Data were analyzed using SPSS version 24.0 for Windows. The descriptive statistics of the dependent variables were tabulated and examined. Alpha level was set to 0.05 for all statistical tests. The Greenhouse–Geisser adjustment for nonsphericity was applied to probability values for repeated measures. Before to analyze data at each post-baseline assessment, a preliminary analysis was carried out to verify whether the groups did not differ in testing parameters. With reference to the global functioning, the two groups showed no statistically significant differences at the baseline in RARS total score and Down’s Scale score, respectively, *t*(25) = 0.81, *p* = 0.42; *t*(25) = 0.20, *p* = 0.83.

With reference to age, the group did not statically differ at baseline, *t*(25) = 0.39, *p* = 0.70. There was no statically significant difference between the two groups in stage variable at the baseline, *t*(25) = 1.20, *p* = 0.77. With reference to the social interactions, the two groups showed no statistically significant differences at the baseline in the classmates’ social ideas and knowledge and the number of social episodes, respectively, *t*(25) = 1.24, *p* = 0.22; t(25) = 0.78, *p* = 0.93. With reference to the motor parameters, such as reaching, touching, grasping and bimanual coordination, the groups did not statistically differ at baseline in each parameter, respectively, *t*(25) = 0.48, *p* = 0.63; *t*(25) = 1.78, *p* = 0.08; *t*(25) = 1.60, *p* = 0.12; *t*(25) = 1.64, *p* = 0.11. With reference to the fine motor skills in school achievement, such as reaching, grasping, maintaining, releasing of school instruments, there were no difference statistically significant between the two group at baseline, respectively, *t*(25) = 2.02, *p* = 0.06; *t*(25) = 1.08, *p* = 0.28; *t*(25) = 1.84, *p* = 0.07; *t*(25) = 1.99, *p* = 0.06. With reference to the cognitive parameters, such as common objects, colors, shapes and measurement concepts, no statistically significant difference between two groups at baseline was found, respectively, *t*(25) = 0.09, *p* = 0.92; *t*(25) = 0.011, *p* = 0.91; *t*(25) = 1.30, *p* = 0.98; *t*(25) = 0.29, *p* = 0.76.

To better understand these data, it is important to note that not statistically significant data do not necessarily indicate a clinical conclusion of an absence of difference. To know whether the observed differences were clinically meaningful from an interval confidence perspective, we reported the confidence interval for the difference in means. For each of the parameters in Table 2, there was no statistically meaningful or statistically significant difference between the group with click4all and the group without click4all, because all the confidence intervals included the null value, zero. Based on these intervals, we did not have enough evidence to conclude that there was a difference in all parameters of interest between the groups at baseline.

**Table 2:** Mean (M), standard deviation (SD) and confidence interval for the difference between the comparison groups at baseline.

Parameters	Group with click4all	Groups without click4all	Difference
	M (SD)	M (SD)	95% CI
Level of RTT severity (RARS)	70.33 (9.19)	67.20 (8.54)	(−4.28, 9.93)
Level of hand functioning (DOWNS’ SCALE)	2.06 (1.27)	2.16 (1.19)	(−1.09, 0.89)
Age	11.60 (10.70)	13.76 (10.75)	(−9.84, 6.70)
Classmates’ social ideas and knowledge (social area)	29.66 (17.67)	22.08 (12.69)	(−4.92, 20.09)
Number of social episodes (social area)	7.40 (2.61)	7.50 (4.01)	(−2.73, 2.53)
Reaching (motor area)	2.73 (1.22)	2.95 (1.17)	(−1.18, 0.73)
Touching (motor area)	2.36 (1.35)	3.20 (1.01)	(−1.81, 0.12)
Grasping (motor area)	1.86 (1.06)	2.58 (1.25)	(−1.63, 0.20)
Bimanual coordination (motor area)	1.10 (0.28)	1.37 (0.56)	(−0.61, 0.06)
Reaching (fine motor area)	2.00 (1.25)	2.95 (1.17)	(−1.93, 0.01)
Grasping (fine motor area)	1.90 (1.08)	2.41 (1.37)	(−1.49, 0.49)
Maintaining (fine motor area)	1.20 (0.41)	1.70 (0.96)	(−1.07, 0.05)
Releasing (fine motor area)	1.00 (0.23)	1.33 (0.219)	(−0.67, 0.01)
Common objects (cognitive area)	2.53 (0.91)	2.50 (1.00)	(−0.72, 0.79)
Colors (cognitive area)	1.46 (0.83)	1.50 (0.67)	(−0.64, 0.57)
Shapes concepts (cognitive area)	1.33 (0.65)	1.33 (0.51)	(−0.55, 0.55)
Measurement concepts (cognitive area)	1.13 (0.51)	1.08 (0.28)	(−0.27, 0.37)

For each parameter, separate analyses of variance (ANOVAs) were carried out. In case of significant effects, paired *t*-test was applied to test whether the scores obtained at T1 were significantly different from those obtained at T2, T3, and T4.

## 3 Results

Table 3 shows means and standard deviation (SD) of social measurements in all phases and in both groups.

**Table 3:** Means (M) and standard deviation (SD) of social measurements in all phases and in both groups.

Groups	Phases	Knowledge	Social episodes
	(T)	M (SD)	M (SD)
Group with click4all	T1	29.67 (1.67)	7.40 (1.61)
	T2	45.67 (1.10)	11.80 (1.96)
	T3	54.67 (1.18)	16.40 (1.23)
	T4	69.67 (1.43)	19.00 (1.59)
Group without click4all	T1	22.08 (1.70)	7.50 (1.01)
	T2	22.08 (1.70)	7.92 (1.52)
	T3	25.00 (1.23)	8.33 (1.68)
	T4	25.42 (1.00)	8.75 (1.22)

**Figure 5:**
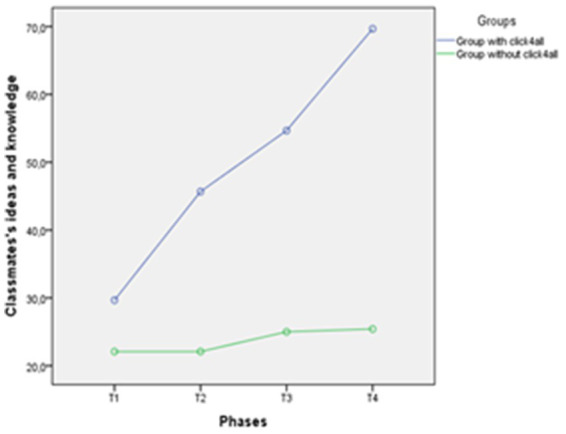
Trend of classmates’ social ideas and knowledge for each phase in the two groups.

A repeated measures analysis of variance was carried out with one between-subjects factor and one within-subjects factor: 2 (groups of participants: group with click4all vs. group without click4all). X 4 (classmates’ social ideas and knowledge in four training phases: T1, T2, T3, T4). Their interaction was also examined. The “group” variable showed a statistically significant effect, *F* (1, 25) = 15.018, *p* < 0.001. This means that the classmates had a different level of social ideas and knowledge according to the two groups. More in depth, classmates showed a high level of knowledge related to participants of the group trained with click4all. In addition, a significant interaction was found in the group × phases, *F*(1, 25) = 37.60, *p* < 0.001. This interaction indicated that the social level of knowledge relating to the two groups showed different trends. The level of ideas and knowledge of classmates related to the group with click4all increased over time, whereas knowledge levels related to the group without click4all were stable over time (see Figure 5). Results from an independent t-test indicated that this difference was significant in T2, T3, and T4, respectively, *t*(25) = 3.98, *p* < 0.001, 95% CI [11.36–35.80]; *t*(25) = 3.81, *p* < 0.001, 95% CI [13.66–45.68]; *t*(25) = 4.72, *p* < 0.001, 95% CI [24.98–63.52]. Based on these confidence intervals, we noted that the difference between the groups was quite large, and it increased over time, indicating that the group with click4ll had a larger level of knowledge about participants with RTT than the group without click4ll at T2, T3, and T4.

**Figure 6:**
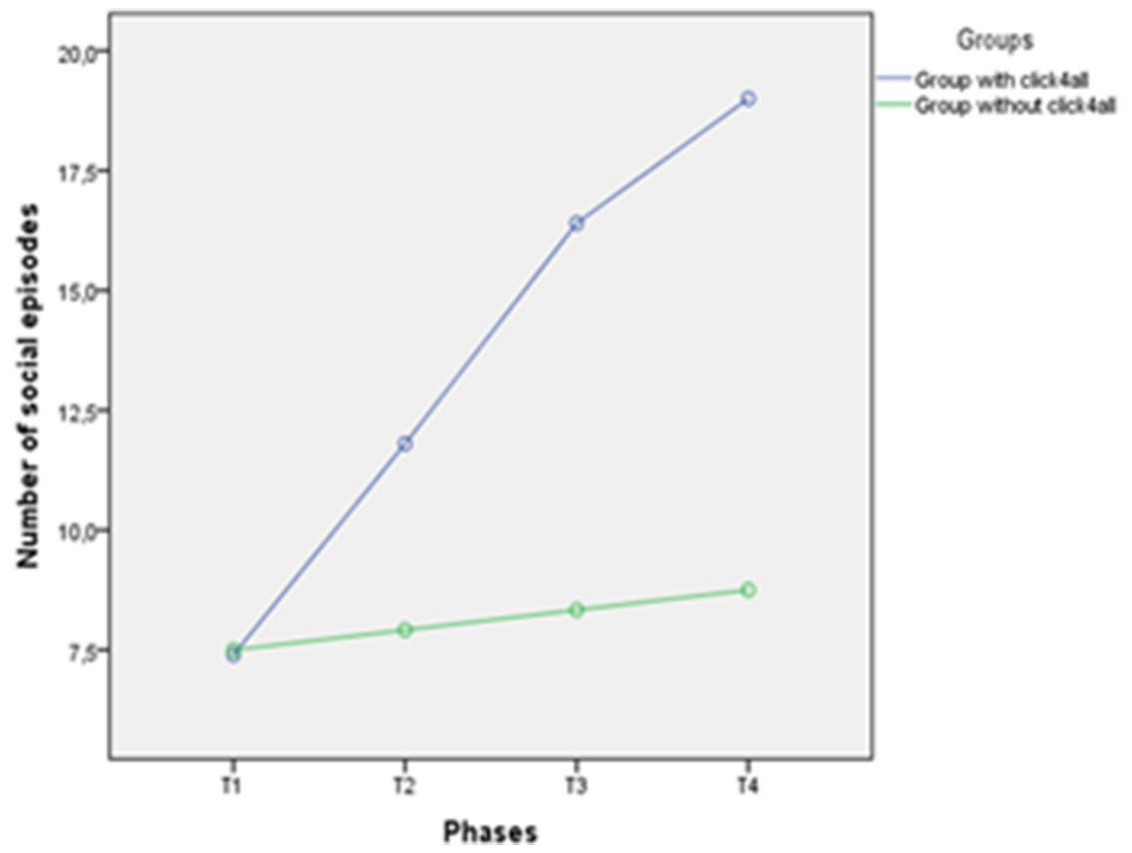
Trend of number of social episodes for each phase in the two groups.

A new repeated measures analysis of variance was carried out with one between-subjects factor and one within-subjects factor: 2 (groups of participants: group with click4all vs. group without click4all) X 4 (number of episodes of social interactions in four training phases: T1, T2, T3, T4). Their interaction was also examined. The “group” variable showed a statistically significant effect, *F*(1, 25) = 8.49, *p* < 0.001. This means that the number of social interactions was higher in the group with click4all than in the group without click4all. There was also a significant interaction group × phases, *F*(1, 25) = 36.10, *p* < 0.001. This interaction indicated that the number of social interactions in the two groups showed different trends. More in depth, social interactions in the group with click4all increased over time, whereas those related to the group without click4all were stable over time (see Figure 6). Results from an independent t-test indicated that this difference was significant in T2, T3, and T4, respectively, *t*(25) = 2.10, *p* < 0.05, 95% CI [0.08–7.68]; *t*(25) = 3.34, *p* < 0.001, 95% CI [3.09–13.04]; *t*(25) = 4.18, *p* < 0.0001, 95% CI [5.20–15.30]. Based on these confidence intervals, we noted that the group with click4all had a higher number of social episodes with participants with RTT at T3 and T4 than the group without click4all. Figure 6 graphically and clearly showed this large difference between groups and time.

Due to the increasing trend of number of social episodes in the group with click4all, to verify what types of social episodes social (eye contact, physical contact and smile) the participants performed and did that changed over time, post-hoc comparisons were carried out (Table 4). As regards eye contact, the results indicated that the number of eye contact between participants with RTT and their classmates significantly increased over the 2 years. Also, the confidence intervals related to this parameter confirmed this trend, because they only contained negative values indicating that the means of the first phases were smaller than that of the other phases. Hence, the eye contact between all participants in the group with click4all increased over time.

**Table 4:** *Post hoc* comparisons of social episodes type (eye contact, physical contact and smile) for each phase (T) in the group with click4all.

Social episodes type	t (p)	95% CI
**Eye contact**		
T1 vs. T2	6.23 (0.0001)	(−8.15, −3.98)
T1 vs. T3	6.75 (0.0001)	(−15.99, −8.28)
T1 vs. T4	6.41 (0.0001)	(−19.74, −9.86)
T2 vs. T3	4.77 (0.0001)	(−8.79, −3.34)
T2 vs. T4	4.59 (0.0001)	(−12.81, −4.66)
T3 vs. T4	2.39 (0.03)	(−5.06, −0.27)
**Physical contact**		
T1 vs. T2	4.34 (0.001)	(−5.67, −1.93)
T1 vs. T3	3.98 (0.001)	(−10.36, −3.10)
T1 vs. T4	3.99 (0.001)	(−13.32, −4.01)
T2 vs. T3	1.69 (0.11)	(−6.62, 0.78)
T2 vs. T4	2.50 (0.25)	(−9.04, −0.69)
T3 vs. T4	1.13 (0.27)	(−5.59, 1.72)
**Smile**		
T1 vs. T2	3.39 (0.004)	(−6.53, −1.47)
T1 vs. T3	4.48 (0.001)	(−4.24, −4.48)
T1 vs. T4	5.95 (0.0001)	(−5.71, −5.95)
T2 vs. T3	2.40 (0.03)	(−0.45, −2.40)
T2 vs. T4	2.99 (0.01)	(−1.40, −2.99)
T3 vs. T4	0.83 (0.42)	(1.26, −0.83)

As regards to physical contact, it was possible to observe a significant increasing trend from T1 to T2, from T1 to T3, and from T1 to T4. Based on the confidence intervals for the comparison between T1 vs. T2, T3 and T4, T3 vs. T4 in physical contact parameter, we observed that the means of the first phases were smaller than that of the other phases. This date indicated that the physical contact increased at T2, T3 and T4. Whereas, for the confidence intervals for the comparisons between T2 vs. T3 and T2 vs. T4 we did not have enough evidence to conclude that there was a difference between these phases.

As regards to smile parameter, it was possible to observe an increasing trend in all the comparisons except for T3 vs. T4. All the confidence intervals (except for T3 vs. T4) indicated that the means of the first phases were smaller than that of the other phases. Hence, the interactions through smiles between the participants with RTT and their classmates increased over time, but this wasn’t observed from T3 to T4.

For each parameter (motor, cognitive and school areas), separate ANOVAs were carried out with one between-subjects factor and one within-subjects factor: 2 (groups of participants: group with click4all vs. group without click4all) X 4 (single parameter in four training phases: T1, T2, T3, T4). Tables 5–7 show means and SDs of motor and cognitive parameters and fine motor skills in school achievement parameters in all phases and in both groups.

**Table 5:** Means (M) and standard deviation (SD) of motor measurements in all phases and in both groups.

Groups	Phases	Reaching	Touching	Grasping	Bimanual Coordination
	(T)	M (SD)	M (SD)	M (SD)	M (SD)
Group with click4all	T1	2.73 (1.22)	2.37 (1.36)	1.87 (1.06)	1.10 (0.28)
	T2	3.30 (1.29)	2.70 (1.37)	2.37 (1.06)	1.37 (0.74)
	T3	3.70 (1.21)	3.60 (1.31)	3.30 (1.01)	1.53 (0.74)
	T4	4.30 (1.37)	4.20 (1.92)	3.77 (1.27)	1.80 (0.98)
Group without click4all	T1	2.96 (1.18)	3.21 (1.01)	2.58 (1.26)	1.38 (0.57)
	T2	3.17 (1.39)	3.29 (1.12)	2.67 (1.39)	1.46 (0.58)
	T3	3.33 (1.30)	3.42 (1.12)	2.96 (1.27)	1.50 (0.64)
	T4	3.54 (1.44)	3.71 (1.23)	3.29 (1.20)	1.63 (0.68)

**Table 6:** Means (M) and standard deviation (SD) of cognitive measurements in all phases and in both groups.

Groups	Phases	Common objects	Colors	Shapes	Measurement concepts
	(T)	M (SD)	M (SD)	M (SD)	M (SD)
Group with click4all	T1	2.53 (0.92)	1.47 (0.83)	1.33 (0.72)	1.13 (0.52)
	T2	2.87 (1.00)	2.20 (1.42)	1.40 (0.91)	1.13 (0.52)
	T3	3.67 (0.90)	3.07 (1.71)	1.93 (1.22)	1.53 (0.93)
	T4	4.13 (0.83)	3.67 (1.40)	2.20 (1.32)	2.03 (0.97)
Group without click4all	T1	2.50 (1.00)	1.50 (0.67)	1.33 (0.65)	1.08 (0.29)
	T2	2.75 (0.75)	1.92 (1.38)	1.42 (0.79)	1.08 (0.29)
	T3	2.92 (0.79)	2.58 (1.08)	1.50 (0.80)	1.25 (0.45)
	T4	3.67 (0.89)	3.00 (1.21)	1.58 (1.00)	1.38 (0.64)

**Table 7:** Means (M) and standard deviation (SD) of fine motor skills in school achievement for all phases and in both groups.

Groups	Phases	Reaching school instruments	Grasping school instruments	Maintaining school instruments	Releasing school instruments
	(T)	M (SD)	M (SD)	M (SD)	M (SD)
Group with click4all	T1	2.00 (1.25)	1.90 (1.09)	1.20 (0.41)	1.00 (0.00)
	T2	2.30 (1.18)	2.17 (1.06)	1.50 (0.63)	1.30 (0.53)
	T3	3.43 (1.13)	2.87 (1.32)	1.87 (0.72)	1.63 (0.64)
	T4	4.17 (1.40)	3.40 (1.39)	2.30 (0.56)	2.03 (0.52)
Group without click4all	T1	2.96 (1.18)	2.42 (1.38)	1.71 (0.96)	1.33 (0.65)
	T2	3.17 (1.39)	2.67 (1.44)	1.88 (0.96)	1.54 (0.72)
	T3	3.33 (1.30)	3.54 (1.25)	2.25 (0.84)	1.83 (0.86)
	T4	3.54 (1.44)	4.08 (1.24)	2.42 (0.63)	2.04 (0.94)

We found no statistically significant differences between the two groups in motor, cognitive and school areas. However, based on partial eta squared we noted both small and moderate magnitude of the differences between groups. More in depth, as regards to motor area, there was a small difference between groups in the reaching parameter, *F*(1, 25) = 0.284, *p* = 0.59, η_p_^2^ = 0.01. As regards to cognitive area, there was a small difference between groups in recognition of colors and shapes parameters, respectively, *F*(1, 25) = 0.619, *p* = 0.44, η_p_^2^ = 0.02; *F*(1, 25) = 0.524, *p* = 0.48, η_p_^2^ = 0.02. Whereas there was a moderate difference between groups in measurement concepts parameters, *F*(1, 25) = 1.412, *p* = 0.25, η_p_^2^ = 0.06. As regards to school area, there was a moderate difference between groups in grasping and maintaining parameters, respectively, *F*(1, 25) = 1.569, *p* = 0.22, η_p_^2^ = 0.06; *F*(1, 25) = 1.860, *p* = 0.18, η_p_^2^ = 0.06.

Even if we found no statistically significant differences, the effect size suggested small and moderate differences between groups indicating that some motor, cognitive and school skills of the group with click4all showed light improvements than those of the groups without click4all.

Due to the broad age range of the sample (5–38 years), we subdivided the two groups by age for median values (7 for the group with click4all and 9 for the group without click4all) and examined the confounding effect of age in outcomes using the Mann–Whitney test with Bonferroni correction (*p* < 0.0016). We found no statistical differences for all parameters and phases. This result showed that age had no role in affecting the outcomes.

We also analyzed the confounding effect of level of RTT severity and the level of hand functionality in outcomes. We subdivided the two groups by RTT severity for median values (67.5 for the group with click4all and 65 for the group without click4all) and by level of hand functioning for median values (2 for both groups). We found no statistical differences for all parameters and phases. This result suggested that both level of RTT severity and level of hand functioning had no confounding effect in the outcomes.

## 4 Discussion

The main aim of this study was to examine whether the click4all, inserted in cognitive and motor training, can increase social interaction of patients with RTT with their classmates in a school setting. Social interactions, motor and cognitive skills and fine motor skills in school achievement were examined in two conditions (training with and without clich4all) over a 2-year period.

The results obtained demonstrated an increase of the number of social episodes over time among classmates and patients with RTT in the group with click4all, compared to the group without click4all. Classmates looked for more interaction with patients with RTT, through physical contact, eye contact and smiles. More in depth, it was found that the number of eye contact between participants with RTT and their classmates increased over the 2 years and in all phases. This is expected data, given that the subjects with RTT mainly communicate through eye contact and gaze (11, 13, 31, 32). However, it is also a significant data because it highlights that the click4all is a valid tool to stimulate eye contact between subjects with RTT and their significant people, such as their classmates. Also, the social interaction through physical contact changed with a trend positive over time, but only in the main phases (from T1 to T2, from T1 to T3, and from T1 to T4). The magnitude of the difference for these comparisons was large, but for the comparisons between T2 vs. T3 and T2 vs. T4 we did not have enough evidence to conclude that there was a difference between these phases. However, this date can be considered not enough relevant from clinical point of view, because the largest differences were observed in other main phases of the training with click4all, as stated above. Also, the social interaction through smile increased in all the comparisons except for T3 vs. T4. Probably, this could occur because eye and physical contact also increased in the last phases of training with click4all, and these two types of social interactions serve a potential communicative function since the clinical features of RTT (31).

With reference to the level of knowledge of classmates about the preference of participants with RTT, we found large differences between groups. In the group with clik4all, the classmates showed a high level of knowledge related to participants with RTT and their social knowledge increased over time. Whereas in the group without click4all the level of social knowledge was stable over time and lower than the group with click4all. Given the social nature of subjects with RTT, the social interactions with typically developing subjects serve as highly motivating experiences. From clinical point of view, it is essential to highlight that, due to the unique nature of RTT, the girls do not adhere to the standard class curriculum. Instead, the focus lies in fostering their involvement within the classroom group and promoting social inclusion. Hence, the findings related to social parameters of this study support the idea that click4all promotes social interactions between subjects with RTT and typically developing subjects in a school setting.

With reference to motor, cognitive and school areas, we found similar improvements in both groups. This was an expected result as the use of click4all aimed to stimulate social interaction and had no clinical purposes. However, even if we found no statistically significant differences between groups, the effect size suggested small and moderate differences indicating that some motor, cognitive and school skills of the group with click4all showed light improvements than those of the groups without click4all.

Taken together, the results of the present study suggest that the click4all can be considered a valid low-tech tool to facilitate and improve the social interactions in subjects with RTT and to reduce the social distance in a school setting. This can be due to the specific features of the click4all. This tool is easily usable and customizable, so it can be easily attractive both for subjects with RTT and typical developing subjects. From clinical point of view, even if the present study had no clinical or therapeutic purposes, we found large and statistically significant difference between groups in the social parameters, but not in motor, cognitive and school parameters. However, in line with Wasserstein (33), it is important to note that not statistically significant data do not necessarily indicate a clinical conclusion of an absence of difference. Considering the magnitude of differences in some motor, cognitive and school parameters, we observed small and moderate differences between groups. This could mean that click4all has a sufficient potential to be developed with therapeutic purposes. Future studies could verify this idea and carry out research in both clinical and school settings.

The results of the current study cannot be discussed in comparison to previous studies because this is the first study that use a low-tech tool to improve social interactions in a school setting. The technological tools used in the previous studies were mainly software for Augmentative and Alternative Communication, microswitches devices or systems for telerehabilitation (11, 34), however no of them were employed in a school setting. Although the technological tools are different, the findings of the present study are in line with that of the previous studies (24), confirming that, overall, the use of digital technologies are valid tools to improve social, cognitive and motor skills of subjects with RTT. However, future research is necessary to identify what low-tech tools can be more adequate for patients with RTT.

The findings of this study must be interpreted in light of two limitations related to the sample size and broad age range of participants. This study involved 27 participants, which is not small for RTT, given that RTT is a rare syndrome. In addition, as RTT is a rare disease, a sample of sufficient cases at the similar age may be hard to obtain. In the present study, the age range of participants is broad (5–38 years) and it can play the role as confounding factor in outcomes. Thus, to verify the effect of age we divided the two groups by age, and we found no statistically significant differences. However, to address these limitations future studies could use a larger sample and different groups ranged in age. Moreover, as RTT is a heterogeneous syndrome with a wide spectrum of symptoms future studies could examine the impact of the click4all in each individual.

The results of this study have practical implications for health professionals, teachers, and researchers. We demonstrated that it is possible to involve classmates in the training for patients with RTT, in a school setting. This result is significant, as patients with RTT do not speak and have complex communication needs, so they are of-ten restricted in a condition of isolation Probably, click4all is a good tool to stimulate social interactions among classmates (35). Moreover, this study showed an increase in social interaction and social knowledge, over a period of 2-years, in the group with click4all. This finding suggests that it is possible to maintain the level of social interactions high over time using a low-tech tool. Hence, the present study supports evidence on how the use of a low-tech tool can stimulate social interaction also with subjects with disabilities (36, 37). However, further research is needed to confirm these findings in other settings and in other NDDs.

## Data availability statement

The raw data supporting the conclusions of this article will be made available by the authors, without undue reservation.

## Ethics statement

The studies involving humans were approved by Ethics Committee of University of Messina. The studies were conducted in accordance with the local legislation and institutional requirements. Written informed consent for participation in this study was provided by the participants’ legal guardians/next of kin. Written informed consent was obtained from the individual(s) for the publication of any identifiable images or data included in this article.

## Author contributions

TC: Formal analysis, Methodology, Supervision, Writing – original draft, Writing – review & editing. LD: Funding acquisition, Resources, Writing – review & editing. MS: Data curation, Investigation, Writing – original draft. ML: Data curation, Investigation, Writing – review & editing. NM: Writing – review & editing, Data curation, Investigation. GZ: Writing – review & editing, Software. RF: Conceptualization, Methodology, Writing – review & editing.
