# Supplementary material for: Use of a low-tech tool in the improvement of social interaction of patients with Rett Syndrome: an observational study
Source: Front Public Health. 2024 Apr 4;12:1353099. doi: 10.3389/fpubh.2024.1353099 (PMC11027742; doi:10.3389/fpubh.2024.1353099)
Supplement: Supplementary file 1 [file Data_Sheet_1.docx]

Appendix A

Description of the typical day of a girl with Rett Syndrome in Italian School

In the educational settings of Italian kindergartens and higher levels of schools, children with disabilities, including those with Rett syndrome (RTT), actively participate in motor activities alongside their neurotypical peers, with necessary facilitations for success. During these activities, the class acknowledges and praises the child's efforts, providing strong positive reinforcement to encourage practice (Romano et al., 2023). Given the social nature of individuals with RTT, these interactions with neurotypical children serve as highly motivating experiences. It is essential to highlight that, due to the unique nature of RTT, the girls do not adhere to the standard class curriculum. Instead, the focus lies in fostering their involvement within the classroom group and promoting social inclusion.

Throughout the school day, a personalized approach is implemented for each girl with RTT. Individualized programs aim to maximize engagement and promote development. These programs include scheduled moments for interaction with classmates, adapting educational activities to facilitate active participation, and managing breaks during the day. A multidisciplinary team, consisting of class and special education teachers, educators, and referral clinicians (therapists and physicians), collaboratively discusses and identifies strategies to enhance the girl's participation.

While the educational dynamics for each girl with RTT are unique and individualized, a general daily schedule can be summarized as follows:

- **Arrival at school:** The day begins with the girl's arrival and a meeting between the caregiver and the special education teacher to exchange information about the girl's status.
- **Morning roll call:** During the morning roll call, the girl responds to her name using methods such as eye-tracking technology, raising a hand, or occasionally using her voice.
- **Girls’ attendance and responses:** This step involves marking attendance and engaging the girl in class activities, such as choosing her preferred food during relevant lessons.
- **Individualized programs:** Programs tailored to the girl's specific needs are implemented throughout the day. For example, tasks may include discriminating between photos or gradually fading assistance in specific activities. Questions given by the child are based on the assessment of Gairs (Fabio et al, 2022).
- **Meeting moments with classmates:** Scheduled interactions with classmates are structured to promote social engagement.
- **Adaptation of educational activities for participation:** Educational activities are adapted and modified to ensure active participation, considering the girl's unique requirements.
- **Snack time:** Designated snack breaks with peers ensure the girls' nutritional needs are met, taking advantage of the social aspect of shared meals.
- **End-of-Day Greeting:** The school day concludes with an end-of-day greeting, providing closure to the day's activities.

This structured approach allows the girl to become an integral part of the classroom environment, fostering social inclusion and overall development. After the educational day concludes at noon, the child has additional time to participate in an active environment, engaging in activities such as walking, playing, and exploring various surroundings.
