# Supplementary material for: Use of a low-tech tool in the improvement of social interaction of patients with Rett Syndrome: an observational study
Source: Front Public Health. 2024 Apr 4;12:1353099. doi: 10.3389/fpubh.2024.1353099 (PMC11027742; doi:10.3389/fpubh.2024.1353099)
Supplement: Supplementary file 2 [file Data_Sheet_2.docx]

QUESTIONNAIRE FOR CLASSMATES

NAME OF STUDENT: _________________ ___________________

Please, answer the following questions about your classmate with Rett Syndrome.

|  | *YES, DEFINE IT* | *NO* |
| --- | --- | --- |
| 1. DO YOU KNOW WHAT HER FAVOURITE CARTOON IS? |  |  |
| 1. DO YOU KNOW WHAT IS HER FAVOURITE SONG? |  |  |
| 1. DO YOU KNOW WHAT HER FAVOURITE COLOR IS? |  |  |
| 1. DO YOU UNDERSTAND IF SOMETHING SHE LIKES OR NOT? |  |  |
| 1. DO YOU THINK SHE CAN LEARN NEW CONCEPTS? |  |  |
| TOTAL SCORE |  | |
